# The effect of combined oral contraceptive pills on angiogenesis in endometriotic lesions

**DOI:** 10.1007/s42000-025-00636-4

**Published:** 2025-02-21

**Authors:** Antonis Siampalis, Efthymia Papakonstantinou, Maria Keramida, Eleftherios Panteris, Sotiris Kalogeropoulos, Neoklis Georgopoulos, Fuminori Taniguchi, George Adonakis, Tasuku Harada, Apostolos Kaponis

**Affiliations:** 1 Dept. of Obstetrics & Gynecology, Patras University School of Medicine, Patras, Greece; 2 Laboratory of Forensic Medicine and Toxicology, School of Medicine, Aristotle University of Thessaloniki, Thessaloniki, 54124 Greece; 3 Dept. of Obstetrics & Gynecology, Tottori University Faculty of Medicine, Yonago, Japan

**Keywords:** Endometriosis, Oral contraceptives, Angiogenesis, VEGF, Endometrioma

## Abstract

**Purpose:**

Neoangiogenesis is necessary for adhesion and invasion of endometriotic lesions. We hypothesize that by blocking angiogenetic pathways we can suppress endometriosis. Oral contraceptive pills (OCs) are routinely used in endometriosis to suppress symptoms of the disease. In the current study, we attempt to evaluate the effects of OCs on various angiogenetic factors in women with endometriosis.

**Methods:**

Sixty women with endometriosis were randomly divided into two groups. Group A consisted of 30 women who received OCs in a cyclical manner for 3 months before surgery and group B of 30 women who did not. Biopsy specimens of ovarian endometrioma were collected. We used qRT-PCR to study the mRNA expression levels of VEGF, TF, PAR-2, SP1, and FGF1.

**Results:**

The levels of mRNA of all angiogenic factors were found to be elevated in women who received OCs compared with women who did not. This difference was statistically significant for VEGF, TF, FGF1, SP1 (*p* < 0.001), and PAR-2 (*p* = 0.046).

**Conclusion:**

OC administration does not inhibit neoangiogenesis in endometriotic lesions; on the contrary, angiogenetic pathways might be upregulated.

**Supplementary Information:**

The online version contains supplementary material available at 10.1007/s42000-025-00636-4.

## Introduction

Endometriosis is a condition in which endometriotic tissue is found outside the uterus (especially in the peritoneal cavity and the ovaries). It affects nearly 10% of women of reproductive age, with pelvic pain and/or infertility characterizing 30–50% of the women [1, 2]. In order for the ectopic endometrium to survive in the peritoneal cavity, a rich vascular network is necessary for oxygen and nutrient supply [1, 3]. The formation of new vessels in endometriotic tissues is mediated by two mechanisms. One is that of formation of new blood vessels from circulating endothelial progenitor cells (EPCs), a process called “vasculogenesis”, and the other is the formation of new vessels from adjacent capillaries upon activation by proangiogenic factors (sprouting angiogenesis) [4, 5]. These two mechanisms commonly coexist and complement each other. The circulating EPCs originate from the bone marrow: they are found in the peripheral circulation and, after being recruited to sites of neovascularization, they differentiate into endothelial cells (ECs) [6]. VEGF and fibroblast growth factor-2 are two of the factors that stimulate migration and proliferation of EPCs [7]. VEGF is a potent activator of angiogenesis, its expression being stimulated by estradiol in endometrial cells during the late proliferative phase [8]. Additionally, it is observed that VEGF is over-expressed in the epithelium of endometriotic lesions [4, 9]. Tissue factor (TF) is a cell membrane-bound glycoprotein that binds to circulating factor VIIa to mediate the activation of both factors IX and X, apart from having a crucial role in hemostasis; it is involved in angiogenesis via intracellular signaling that utilizes the protease activated receptor-2 (PAR-2) [10, 11]. Transcription factor specificity protein 1 (Sp1) is thought to regulate VEGF expression in several carcinomas, such as pancreatic adenocarcinoma and ovarian cancer; it also regulates the expression of matrix metalloproteinases (MMPs), a group of zinc-dependent protein and peptide hydrolases, which contribute to angiogenesis via proteolytic degradation of the extracellular matrix (ECM) and the vascular basement membrane, thereby enhancing the invasion of ECs into the surrounding tissues [12, 13]. Fibroblast growth factors (FGFs) -which are local angiogenic stimulator molecules-, and specifically FGF-1 and FGF-2, are involved in EC activation in endometriotic lesions and are over-expressed in endometriosis, having a role in angiogenesis as well as endometriotic cell invasiveness [14].

Given that endometriosis is a recurrent disease, medical treatment should aim to control the symptoms rather than cure the disease [15]. Since ovulation and menstruation play a major role in the pathogenesis of endometriosis, an easy and effective therapeutic approach seems to be that of suppression of menstruation and ovulation via hormonal treatments, such as oral contraceptives (either combined or progestin-only) and GnRH analogs [16]. Examination of the effect of the combined oral contraceptive pill (COCP) to control endometriotic lesions in the setting of endometriotic-associated angiogenesis is thus of great interest since the formation of new vessels plays a major role in the pathogenesis of endometriosis.

Our aim is to study the above relationship using a real time PCR method to evaluate the expression of VEGF, PAR-2, TF, FGF1, and SP-1 factors at the level of mRNA in endometriotic lesions in women who have received COCP treatment and those who have not.

## Materials & methods

The subjects in this study were women of reproductive age. From January 2017 to December 2023, 60 women with known endometriosis (stages 2 and 3) were recruited. The staging of endometriosis was based on the rASRM classification system [17]. Stage 2 includes women with ovarian endometrioma and superficial ovarian endometriosis, peritoneal filmy adhesions, or deep peritoneal endometriosis. Stage 3 includes women with ovarian endometrioma, deep peritoneal endometriosis with dense adhesions, and partial obliteration of the cul de sac. Their mean age was 38 years. They were nulliparous and had a mean body mass index (BMI) of 27 kg/m^2^. The ovarian endometrioma present in all participants was diagnosed using ultrasonography and/or magnetic resonance imaging. Women with perimenopausal symptoms such as hot flashes, night sweats, and/or irregular menstrual periods were excluded from the current study. The inclusion and exclusion criteria are presented in Table 1.

**Table 1:** Inclusion and exclusion criteria

INCLUDED	EXCLUDED
Reproductive age (mean age 38 years)	Perimenopausal symptoms (*hot flashes*,* night sweats*,* irregular menstrual bleeding)*
Personal history of infertility	FSH > 12 IU/dl*
Mean BMI 27 kg/m^2^	Obesity (BMI > 30 kg/m^2^)
Endometriosis stage II or greater	Hormonal treatment (< 12 months before surgery)
Endometriomas > 5 cm	

*Measured on 2nd day of menstrual period

This was a randomized follow-up study with analysis of ovarian samples derived from OC-treated (ethinyl-estradiol and dospirenone, Bayer Hella, ABEE) and non-OC-treated women before surgery. The randomization was performed by accessing a central internet-based randomization program, MinimRan [18]. The random allocation sequence and the assignment of the participants to interventions were made by two of the authors (A.K. and S.K).

After enrollment, the women were randomized into two groups (Table 2). Group A (OC+) consisted of 30 women with a mean age of 35.5 years and a mean BMI of 27 kg/m^2^. Seventeen of them had stage 2 and 13 had stage 3 endometriosis. They received OC for a period of 3 months prior to surgery and had not been administered any hormonal treatment within the 12 months before the surgical procedure. Group B (OC-) consisted of 30 women with a mean age of 38 years and a mean BMI of 27 kg/m^2^. Sixteen of them had stage 2 and 14 had stage 3 endometriosis. They did not receive OC treatment before surgery. In addition, no other hormonal therapy had been administered within 12 months prior to surgery.

**Table 2:** Stage of endometriosis

	Oral contraceptives	
	No	Yes
	*N*	*N*
Duration of treatment (months)	-	3
Stage 2 enometriosis	16	17
Stage 3 endometriosis	14	13

During laparoscopy, biopsy specimens of the ovarian endometrioma were collected. A sample of the endometrioma cyst wall, in the distal part of the cyst, was collected in all cases. In group B, surgery was performed during the proliferative phase of the menstrual cycle. All biopsy specimens were collected in accordance with the guidelines of the Declaration of Helsinki and with the approval of the ethical committee of the General University Hospital of Patras. Informed consent was obtained from all women.

### qRT-PCR

Quantitative real-time polymerase chain reaction (qRT-PCR) is used to study the expression of genes in various tissues. This method is one of the most common tools, enabling relative quantification of target gene expression by comparison with the expression of a reference or a housekeeping gene. A housekeeping gene is defined as being constitutively expressed in the tissue under study [19]. The reference gene should have stable expression under all experimental conditions (i.e., patients and controls) and be expressed appropriately in the tissue studied, otherwise results may be biased.

### For primer design

The gene sequences of the exons and introns used for the design of the specific primers were obtained from the ensemble database (EMBL-EBI) (Online Resource 1). Primer design, purchased from Thermo Fisher Scientific, using the gene sequences was performed with the NCBI tool Primer-Blast, according to the instructions of the manufacturer. The following criteria were considered in the development of the primers [20]:

1. Length of 18–24 bases.
2. 40–60% G/C content.
3. Starts and ends with 1–2 G/C pairs.
4. Melting temperature (TM) of 50–65 °C.
5. The two primers of a primer pair should have closely matched melting temperatures for maximizing PCR product yield.
6. Primer pairs should not have complementary regions.
7. The amplicon length is dictated by the experimental goals. For qPCR, the target length is closer to 100 bp and for standard PCR it is near 500 bp (Online Resource 2; Online Resource 3).

Fresh tissue samples were cut < 0.5 cm and immersed in 5–10 volumes of RNAlater Stabilization Solution (Invitrogen, Cat. No. AM7020), stored at 4 °C overnight, and then moved to − 80 °C until RNA extraction for long-term storage.

### Tissue lysis and RNA extraction

Prior to RNA isolation, the samples were lysed and homogenized. The frozen tissue was placed on ice and 0.5 mL of TRIzol Reagent (TRIzol Reagent, Cat. No: 15596026, Invitrogene) was added at optimal sample size (50–70 mg) and homogenized at 25 Hz for 3 min. 0.1 mL of chloroform was added to 0.5 mL of Trizol reagent, shaken vigorously by hand for 15 s, and incubated at room temperature for 3 min. The samples were centrifuged at 11.600 x g for 15 min at 4 °C. An equal volume of ice-cold 75% ethanol was added to the upper phase and transferred to a High Pure Filter Tube of the High Pure RNA Isolation Kit (Cat. No. 11 828 665 001, Roche) [21]. RNA isolation was performed according to the isolation kit protocol [22]. The concentration and purity of RNA was determined by measuring the absorbance at 260 nm and 280 nm in a spectrophotometer. The yield of total RNA was 0.5–0.8 µg/mg.

DNA (cDNA) synthesis was performed with a mixture of anchored-oligo (dT) primers and 1 µg of total RNA, according to the manufacturer’s instructions (Transcriptor First Strand cDNA Synthesis Kit, Cat. No. 04897030001; Roche Applied Science). Real-time PCR was carried out in the LightCycler 2 Instrument (Roche) using the FastStart Universal SYBR Green Master (Roche Hellas).

Four independent experiments were analyzed in duplicates for all data shown. GAPDH was used as a reference gene for normalization. To analyze qPCR data, REST-MCS beta software version 2 was used.

### Statistical methods

Data were analyzed using IBM SPSS v26 (IBM Corp. Released 2019. IBM SPSS Statistics for Windows, Version 26.0. Armonk, NY: IBM Corp). As parameters did not follow a normal distribution (assessed using the Shapiro-Wilk normality test), a non-parametric Mann-Whitney U test was used to determine statistical differences between the two groups. Two-tailed p-values < 0.05 were considered statistically significant. Gene expression level values are presented as log2 fold change (median [min-max]) relative to untreated control after normalization against GADPH mRNA levels, which was used as an internal control. A statistically significant increase in log2 transformed fold change values was observed in the relative mRNA expression of all genes.

## Results

A sample of 60 women, 30 treated with oral contraceptives, and 30 controls without treatment in the proliferative menstrual cycle phase, participated in this study. The patients did not have demographic differences as age and BMI were similar in the two study groups. Median values with minimum and maximum are presented, as follows: age was 35 (22–49 years) for the control group and 37 (21–49 years) for the experimental group (*p* = 0.711). BMI was 27 (25–29) in both groups (*p* = 0.561). Table 3 presents the demographics for each group.

**Table 3:** Demographic parameters and angiogenetic factors gene expression between women who ’ did not receive OC and women who received OC

	Oral Contraceptives		
	No	Yes	*p* -value *
BMI	27 (25, 29)	27 (25, 29)	0.561
AGE	35 (22, 49)	37 (21, 49)	0.711
TF	3.20 (2.70, 4.50)	4.04 (2.31, 6.16)	< 0.001
PAR-2	7.65 (6.90, 9.00)	9.54 (3.21, 15.64)	0.046
SP1	1.57 (0.98, 2.10)	10.88 (2.08, 17.78)	< 0.001
VEGF	1.52 (0.90, 2.30)	11.37 (0.64, 22.03)	< 0.001
FGF1	1.50 (0.80, 2.10)	3.60 (2.10, 7.10)	< 0.001

*Mann–Whitney U test

Quantitative variables are presented as median (min, max)

**Fig. 1:**
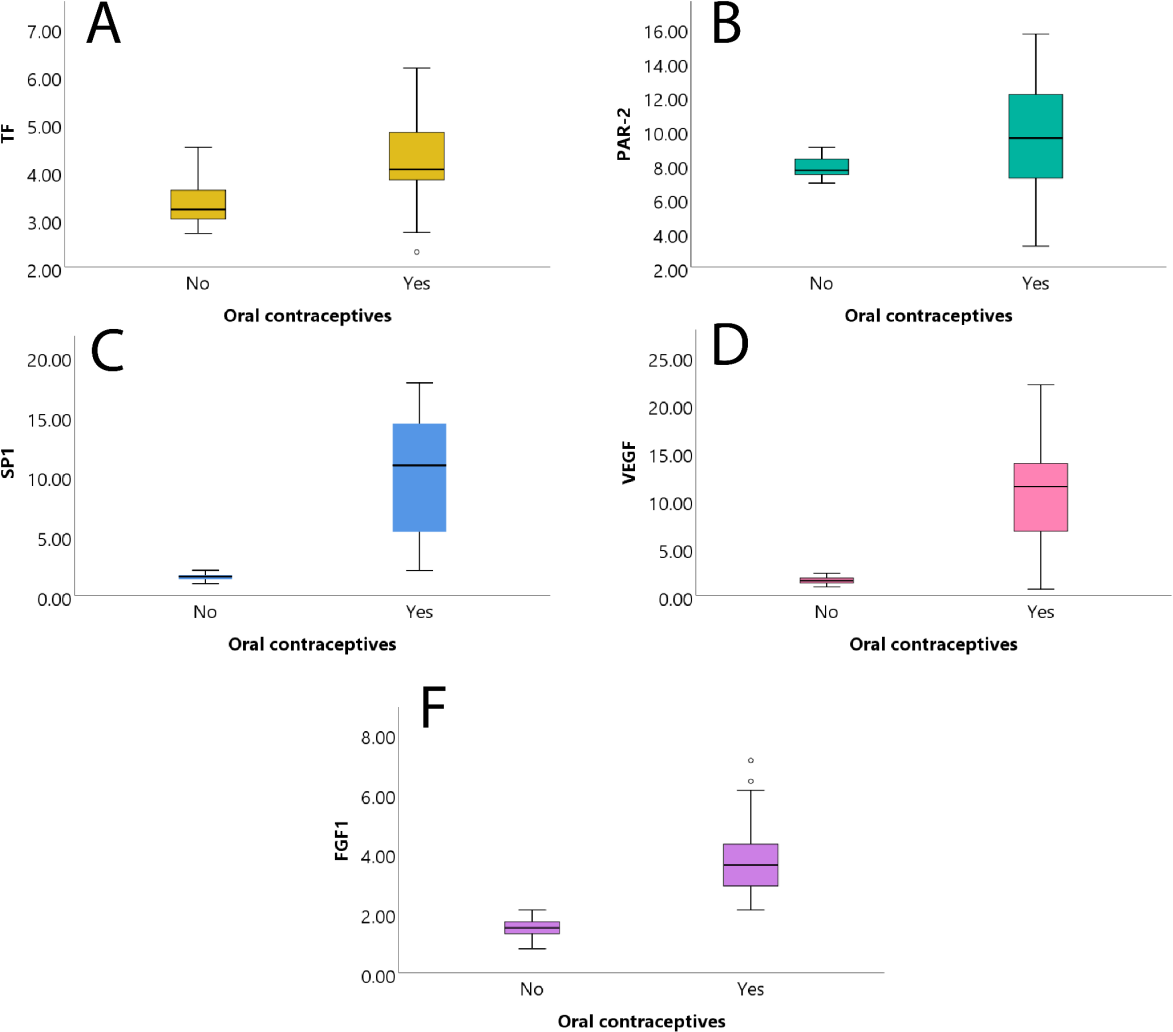
Box plots with relative mRNA Log2 expression values for the two study groups. (**A**) Tissue factor with and without oral contraceptive (OC). (**B**) Protease-activated receptor 2 (PAR-2) with and without OC. (**C**) Specificity protein 1 (SP1 transcription factor) with and without OC. (**D**) Vascular endothelial growth factor (VEGF) with and without OC. (**F**) Fibroblast growth factor 1 (FGF-1) with and without OC

Median log2 transformed fold change expression levels of the relevant angiogenic factors were statistically differentiated between the two groups. Angiogenic factors were significantly increased after the treatment with oral contraceptives. In detail, TF expression was 3.20 (2.70, 4.50) vs. 4.04 (2.31, 6.16) in the control versus the experimental groups, respectively (*p* < 0.001). Similarly, PAR-2 expression was 7.65 (6.90, 9.00) vs. 9.54 (3.21, 15.64) in control versus experimental groups, respectively (*p* = 0.046). SP1 expression was also significantly differentiated, with 1.57 (0.98, 2.10) vs. 10.88 (2.08, 17.78) in control versus experimental groups, respectively (*p* < 0.001). VEGF expression was highly differentiated, with 1.52 (0.90, 2.30) vs. 11.37 (0.64, 22.03) in control versus experimental groups, respectively (*p* < 0.001). Finally, FGF1 was differentiated with 1.50 (0.80, 2.10) vs. 3.60 (2.10, 7.10) in control versus experimental groups, respectively (*p* < 0.001) (Table 3; Fig. 1).

## Discussion

COCPs, containing ethinyl-estradiol (EE) and progestins given cyclically or continuously have long being used for the treatment of endometriosis. They are effective in ameliorating endometriosis-associated pain and have been used post-surgery to prevent recurrence. They are one of the first line symptomatic treatments for women suffering from this condition [15, 23].

In the current study, we evaluated the effects of COCP administration on various angiogenic growth factors in women with endometriosis. Recently we showed that GnRH-a administration decreased the mRNA expression of VEGF-A, TF, and PAR-2 in endometriotic tissues from women with endometriosis [24]. Working upon this notion, we hypothesized that COCPs can also decrease the expression of angiogenic factors in women with endometriosis. On the contrary, we obtained the exact opposite result, observing that COCP administration given cyclically for 3 months significantly increases the mRNA expression of VEGF-A, TF, SP1, and FGF1 with a moderate increase of PAR-2. This might be due to the effect of estradiol on angiogenetic factors.

In women without endometriosis, estrogens increase the expression of angiogenic factors, whereas progesterone (P4) alone exerts a variety of angiogenic or antiangiogenic effects, with various influences on neovascularization. Both ovarian sex steroids, estradiol (E2) and progesterone (P4), play a role in angiogenesis in the endometrium, this being demonstrated in the difference in VEGF expression in the proliferative vs. the secretory phase [25]. VEGF expression has been found to be enhanced in the late proliferative and early secretory phase vs. the early proliferative phase, while in the late secretory phase, VEGF was moderately expressed, an event that correlated with the absence of E2 and P4 [25]. Similarly, another research work demonstrated lower VEGF mRNA expression during the secretory phase, correlating this finding with the presence of progesterone and the absence of estrogens [27]. When cultures of endometrial stromal cells were added to medroxyprogesterone acetate, the release of VEGF was inhibited [26]. Notably, mid-secretory expression of VEGF receptor is considered necessary for the proper implantation of the embryo [26]. Our results demonstrate that COCPs not only do not suppress mRNA VEGF-A expression but, in fact, upregulate it, since all the women we studied were in the proliferative phase. This is the first time, as far as we know, that the expression of VEGF-A mRNA has been studied in endometriomas after COCP use; the only bibliographic reference that has studied VEGF protein expression after COCP use using immunohistochemistry was in the endometrium of patients with myomas and menorrhagia [26]. In these patients, aged 35–49, VEGF protein expression was found to be diminished compared to that of patients in the proliferative phase and without a history of COCP use [27]. In general, it may be hypothesized that this result is due to the effect of progesterone rather than to that of estradiol.

While oral contraceptives (OCs) often reduce VEGF expression in the normal endometrium, endometriotic lesions present a more complex environment. One research paper has shown that 17β-estradiol drives VEGF via the Wnt/β-catenin pathway [27], this observation being supported by other studies highlighting the importance of hormone-Wnt signaling in reproductive tissues [28, 29]. However, endometriotic cells frequently exhibit progesterone resistance partly because of altered progesterone receptor expression and function [30–32]. This lack of responsiveness is further amplified by the chronic inflammatory state typical of endometriosis where cytokines such as IL-6 and TNF-α can work in tandem to elevate VEGF levels [1, 33]. Additionally, mounting evidence indicates that local steroid metabolism and nuclear receptor signaling diverge significantly in endometriotic lesions compared to the eutopic endometrium, altering how these tissues respond to OCs [34, 35]. As Bulun notes, the modified endocrine microenvironment in endometriosis can counteract progestin’s usual suppression of angiogenic factors [36]. Taken together, these findings explain why, in contrast to the inhibitory effect of OCs on VEGF in the eutopic endometrium, endometriotic lesions might maintain or even raise VEGF production.

In addition to its role in the hemostatic cascade, TF is involved in angiogenesis through signaling that utilizes the protease-activated receptor 2 (PAR-2) [37]. In previous studies, TF was found to be increased in the blood of women treated with COCPs, more specifically in monocytes [38]. In another study, antiprogestin RU486 was observed to inhibit the progestin-enhanced transcription of pTF 278 and pTF 111 promoter constructs; moreover, several studies have reported that TF is persistently upregulated in human endometrial stromal cell decidualized by progestins [39]. Our results are in accordance with those of the above studies since the administration of COCPs significantly increased the expression of TF mRNA focally on endometriomas.

PAR-2 contributes to the progression of endometriosis according to several studies [40]. PAR-2 is present on human vascular ECs and mediates EC proliferative responses when activated [41]. In a study published in 2014 in a mouse xenograft model of human endometriosis, PAR-2 inhibitor ENMD-1068 dose-dependently inhibited endometriotic lesion development and IL-6 and NF-κβ expression [40]. In our study, as regards PAR-2 mRNA expression after COCP use in woman with endometriosis, a slight decrease in expression was noted between OC + and the control group. In contrast, GnRH-a was found to significantly suppress PAR-2 expression in women with endometriomas [24]. Our findings have confirmed the fact that COCPs do not suppress endometriosis development and alleviate endometriosis-related symptoms solely via other mechanisms.

Specificity protein 1 (Sp1) is an important transcription factor that regulates many critical biological functions, including cell proliferation, apoptosis, invasion, and metastasis by binding to the promotor regions of its target genes. Previous studies have demonstrated that Sp1 is aberrantly expressed and plays important roles in cancer by stimulating the growth of tumor cells. However, the expression and role of Sp1 in endometriosis remains unknown.

SP1 is a transcription factor that regulates numerous functions, such as cell proliferation, apoptosis, and invasion [42]. Investigators have found that SP1 mRNA and protein are highly expressed in the ectopic endometrium compared with the normal endometrium, thus verifying that in endometriosis SP1 is upregulated [42]. Based on the above data, we studied the effect of COCPs on SP1 expression in endometriotic tissues and found that SP1 was even higher in the OC + group than in the control group. It appears that not only is SP1 expression not affected by COCP use but it is further enhanced. On the other hand, as recently published, GnRH-a has no effect on SP1 expression in endometriotic tissues [24].

The receptor FGFR1 is expressed in vascular ECs, its activation leading to activation of FGFR3 in the endothelium [14]. FGF signaling is hypothesized to influence the entire process of angiogenesis, while up-regulation of FGFR1 and FGFR2 promotes angiogenesis and vascular endothelial proliferation [43]. Extracellular matrix degradation, an important step of angiogenesis, is promoted by several FGFs, including FGF1, via upregulation of MMP expression in ECs [43]. That is why we chose FGF1 as an important protein and studied its gene expression in endometriosis while exploring how COCPs might affect it. Our results demonstrated a higher expression in the OC + group than in the control (OC-) group. This demonstrates that FGF1 expression is likely to be hormone-dependent and should be of concern to gynecologists treating women who suffer from endometriosis with COCPs since their role in angiogenesis is questionable.

The present study is of high originality given the fact that it is the first time, to the best of our knowledge, that a study has explored the impact of COCP treatment preoperatively on angiogenetic pathways in women with endometriosis. One disadvantage of the present study is that we examined only the mRNA expression of TF, PAR2, VEGF, SP1, and FGF1 and not their protein expression using Western blot, ELISA, or immunohistochemistry. COCPs do not clearly inhibit neoangiogenesis in endometriotic lesions; on the contrary, some angiogenetic pathways may be upregulated. Further research should be conducted to discover new, more effective treatments for endometriosis, a common disease that affects many women of reproductive age, and identifying ways to affect its angiogenesis holds much promise in this direction.

## Electronic supplementary material

Below is the link to the electronic supplementary material.

Supplementary Material 1
