# Correction: The effect of combined oral contraceptive pills on angiogenesis in endometriotic lesions

**DOI:** 10.1007/s42000-025-00715-6

**Published:** 2025-09-17

**Authors:** Antonis Siampalis, Efthymia Papakonstantinou, Maria Keramida, Eleftherios Panteris, Sotiris Kalogeropoulos, Neoklis Georgopoulos, Fuminori Taniguchi, George Adonakis, Tasuku Harada, Apostolos Kaponis

**Affiliations:** 1 Department of Obstetrics & Gynecology, Patras University School of Medicine, Patras, Greece; 2 Laboratory of Forensic Medicine and Toxicology, School of Medicine, Aristotle University of Thessaloniki, Thessaloniki, 54124 Greece; 3 Department of Obstetrics & Gynecology, Tottori University Faculty of Medicine, Yonago, Japan

**Hormones (2025) 24:517–524**

10.1007/s42000-025-00636-4

In the original version of this article, the given and family names of all authors were incorrectly structured.

The original article has been corrected.

