# Supplementary material for: The effect of combined oral contraceptive pills on angiogenesis in endometriotic lesions
Source: Hormones (Athens). 2025 Feb 21;24(2):517–24. doi: 10.1007/s42000-025-00636-4 (PMC12339599; doi:10.1007/s42000-025-00636-4)
Supplement: Supplementary file 1 — Supplementary Material 1 [file 42000_2025_636_MOESM1_ESM.docx]

**Supplementary material**

**Table 1** Genes Sequences Transcript ID

| Genes Sequences | Transcript ID |
| --- | --- |
| Sp1 gene (Sp1 transcription factor) | ENST00000327443.9 |
| VEGFA gene (vascular endothelium growth factor A) | ENST00000372055.9 |
| PAR-2 gene/F2RL1 (F2R like trypsin receptor 1) | ENST00000296677.5 |
| FGF-1 gene (fibroblast growth factor 1) | ENST00000337706.7 |
| F3 gene/coagulation factor III/tissue factor (TF) | ENST00000334047.12 |

**Table 2**  Oligonucleotide primers used in quantitative RT-PCR (real-time polymerase chain reaction)

| Gene | Forward primer | Reverse Primer |
| --- | --- | --- |
| Sp1 | 5'-GACCCCCTTGAGCTTGTCCCT-3' (21b) | 5'-CTGTGAAAAGGCACCACCACC-3' (21b) |
| VEGFA | 5'-TTGCCTTGCTGCTCTACCTCCA-3' (22b) | 5'-GATGGCAGTAGCTGCGCTGATA-3' (22b) |
| PAR-2 | 5'-CTGAGTTTCGAATCGGCGG-3' (19b) | 5'-GGATGTGCCATCAACCTTACC-3' (21b) |
| FGF-1 | 5'-CAGTGGATGGGACAAGGGAC-3' (20b) | 5'-GGTTCTCCTCCAGCCTTTCC-3' (20b) |
| TF | 5'-AGACAGCCCGGTAGAGTGTATG-3' (22b) | 5'-TGCCCCACTCCTGCCTTTCTAC-3' (22b) |
| GAPDH | 5'-CCATGTTCGTCATGGGTGTGA-3' (21b) | 5'CATGGACTGTGGTCATGAGT-3' (20b) |

**Table 3** Tm of selected primers

| Primer | Tm (Melting Temperature) |
| --- | --- |
| SP1F | 63,81 |
| SP1R | 61,96 |
| VEGFAF | 63,93 |
| VEGFAR | 62,79 |
| PAR2F | 59 |
| PAR2R | 58,98 |
| FGF1F | 60,4 |
| FGF1R | 60,03 |
| TFF | 61 |
| TFR | 64 |
| GAPDHF | 60,61 |
| GAPDHR | 57,22 |
